# Clinical characteristics of headache in Italian adolescents aged 11–16 years: a cross-sectional questionnaire school-based study

**DOI:** 10.1186/s13052-018-0486-9

**Published:** 2018-04-04

**Authors:** Thomas Foiadelli, Alessandra Piccorossi, Lucia Sacchi, Mara De Amici, Maurizio Tucci, Ilaria Brambilla, Gian Luigi Marseglia, Salvatore Savasta, Alberto Verrotti

**Affiliations:** 1 Department of Clinical-Surgical, Diagnostic and Pediatric Sciences, University of Pavia, Pediatric Clinic, I.R.C.C.S. Policlinico “S. Matteo” Foundation, 27100 Pavia, Italy; 2 Department of Pediatrics, University of L’Aquila, San Salvatore Hospital, L’Aquila, Italy; 3 Department of Electrical, Computer and Biomedical Engineering, University of Pavia, Pavia, Italy; 4 Laboratorio Adolescenza association, Pavia, Italy

**Keywords:** Adolescence, Headache, Migraine, Population-based, Questionnaire, Italian adolescents, Self-medication, Quality of life, Public health

## Abstract

**Background:**

The purpose of this study was to determine headache characteristics, impact on daily activities and medication attitudes among a large sample of adolescents in Italy.

**Methods:**

Secondary school classes were randomly selected from a national stratified multistage sampling. Data regarding socio-familial factors, headache characteristics, impact on daily activities and medication use were recorded with an anonymous multiple-choice questionnaire.

**Results:**

The survey involved 2064 adolescents. 1950 questionnaires were considered for analysis. Study population included 944 males (48.4%) and 1006 females (51.6%), aged between 11 and 16 years (mean 13.5 ± 1.87). Headache prevalence was 65.9%. Mean age at headache onset was 8.33 years. 9.8% suffered from headache > 1/week, 14.3% > 1/month, 24.2% monthly and 17.7% less than monthly. The mean duration of a headache episode was less than 30 min in 32.9%, 1 hour in 28.1%, 2 hours in 19.3% and several hours in 19.5%. Pain intensity was moderate in 52.2% and severe in 9.5%. School represented the main trigger factor (67%). Impact on daily activities was noted in 57.5%. 69.2% of adolescents reported the use of pain relievers. Up to 5.7% declared self-medication, while only 20.6% followed a physician’s prescription. Female adolescents experienced headache more frequently (70.2% vs 60%) and more intensely than male peers. Girls had a higher family history of headache, could more frequently identify a trigger factor, and were more affected into their daily activities than boys.

**Conclusions:**

Population-based studies of headache disorders are important, as they inform needs assessment and underpin service policy for a disease that is a public-health priority. Headache has a high prevalence among adolescents and carries a significant burden in terms of impact on daily activities and use of medication. Furthermore, underdiagnose is common, while trigger factors are often detectable. Special consideration should be given to female adolescents and self-medication attitudes.

## Background

Headache represents a common health problem in the pediatric age. By affecting daily activities, school performance and social relationships (i.e. with family and friends), headache can negatively impair quality of life, as well as physical and mental health. Several triggers and risk factors such as lifestyle, environment and psychological or psychiatric aspects can influence headache prevalence and frequency [1, 2]. Moreover, different comorbidities including behavioral problems, mood disorders (i.e. anxiety and depression), allergies, chronic diseases such as asthma and obesity, and sleep disorders can play a role in the pathophysiology of headache in childhood [2].

Numerous studies have assessed headache prevalence in children and adolescents in different geographic areas and settings (i.e. epidemiological population studies [3–10] or clinical tertiary centers-based surveys [11, 12]). Headache in the pediatric population is variously reported, ranging between 2.8 and 91%, with a mean overall prevalence of 58.4% [13]. Many studies have highlighted an increasing incidence of primary headache and migraine among school-aged children in the last decades [14]. Furthermore, a gradual increase of headache prevalence during the transition from preschool to elementary school has been reported [15]. Headache prevalence reaches a peak in adolescence, acquiring the characteristic higher female-male ratio after this period [14]. Finally, chronic evolution into adulthood is possible: a longer time between headache onset and first medical consultation has been correlated with a worse outcome, stressing the importance of adequate diagnosis and management in young patients [16].

As emphasized by the Global Burden of Disease Study 2010 from the Institute of Health Metrics and Evaluation, population-based studies of headache disorders are important, as they inform needs assessment and underpin service policy for a disease that is a public-health priority [17]. Nevertheless, only few population-studies have tried to determine headache prevalence among adolescents in Italy in the past years [1, 3–5, 11], the vast majority focusing on migraine or very selected populations from tertiary centers for headache management. The aim of this study was to fill this gap, by describing headache characteristics and impact on quality of life in a large group of Italian adolescents, with a detailed analysis of trigger factors, familiar and social co-factors, self-perceived pain evaluation, and medication attitudes.

## Methods

### Study population

This cross-sectional study was conducted between November 2015 and April 2016. It involved a nationally representative sample of 2064 adolescents attending the 6th, 7th and 8th grade of secondary school (1016 boys and 1048 girls). Data were collected from the survey "Teenagers and Headache" in the context of the Survey On Lifestyles of Teenagers conducted by the “Italian Society of Adolescent Medicine” and the “Adolescence Laboratory” Association, in collaboration with the Pediatric Departments of the University of Pavia and L’Aquila University.

Random selection of school classes was performed on the basis of a national stratified multistage sampling (data from the Italian Ministry of Education University and Research (MIUR) for secondary schools, first degree), according to a factorial design that considered the geographic distribution (North-East; North-West, Central Italy, South, Islands) and, within each area, the size of the population.

### Data collection

During school hours, in the presence of the teacher, a multiple-choice questionnaire was administered to each student. Instructions for completing the form were provided by the teachers in charge of the students. The questionnaires were anonymous and no personal information was recorded.

The first section of the questionnaire consisted of socio-demographic data, family background and family history of headache. In the second section, questions concerned headache characteristics and frequency, age of onset, pain intensity and mean duration, seasonality and day/night occurrence, presence of trigger factors, and impact on daily activities. Pain intensity evaluation was based on a comparative age-appropriate pain assessment scale that combined a numerical rating with a verbal descriptor scale, as follows: mild (1–3), moderate (4–6), severe (7–10). The third section of the survey concerned medication use, including timing, criteria for drug choice, self-medication and perceived worries about the use of analgesics. Prophylactic treatment was not considered in the medication section of questionnaire.

Students were invited to fill in all the sections of the questionnaire, were applicable.

According to the Italian laws and good ethical practice, a written and informed consent of the parents was required prior to participation to the study. The questionnaire and the study protocol were accepted by the Ethical Committee of the Medical Faculty of the University of Pavia.

### Statistics and data analysis

Data analysis was performed using IBM SPSS version 20 and MatlabR2016a (The Mathworks, Inc). Quantitative variables were summarized as mean and standard deviation (SD) and categorical variables were summarized as a number (%). Distributions of categorical variables between subgroups were compared using the chi-square test, whereas the Wilcoxon test was used to compare quantitative variables. Statistical significance was considered for all tests at *p* < 0.05.

### Headache characterization

To define the headache prevalence in our study, the population was divided in two groups based on the answer to the question “How often do you experience headache?”: one group including students reporting headache at least several times a year, from almost daily to less than monthly (Group 1), and another one including those having suffered from headache very rarely (less than 5 episodes lifelong) or never (Group 2). Further subanalysis focusing on headache characteristics, medication use, gender and age-related differences, social and familial factors were performed on Group 1.

## Results

### Characteristics of the study population

A total of 2064 questionnaires were distributed. One-hundred and fourteen questionnaires lacked parental consent and were therefore excluded. The remaining 1950 questionnaires were considered appropriate for data analysis. The examined sample included 944 males and 1006 females (48.41% vs 51.59%). Age ranged between 11 and 16 years (13.5 ± 1.87). The vast majority of the students was born in Italy (94.44%) from Italian parents (83.77%), while a rate of 9.02% had both non-Italian parents. The participant’s family situation was as follows: adolescents living with both parents (83.21%), living with only one parent (11.28%), living with one parent and a stepmother/stepfather (4.89%), and living without parents (0.57%).

Table 1 describes the general characteristics of the study population.

**Table 1:** Demographic data and socio-familial factors

	Overall	Group 1	Group 2	Sign
	(n = 1950)	(*n* = 1272)	(*n* = 678)	(*p* value)
Gender (*n* = 1950)				
Males	944 (48.41%)	566	378	< 0,001
Females	1006 (51.59%)	706	300	
Age (*n* = 1937, 13 missing)				
11–12 years	71 (3.66%)	49	22	0,9
13–14 years	1830 (94.48%)	1189	641	
15–16 years	36 (1.86%)	25	11	
Place of birth (*n* = 1944, 6 missing)				
Italy	1836 (94.44%)	1192	644	0,145
Abroad	108 (5.56%)	78	30	
Geographic area (*n* = 1951)				
North-West	460 (23.58%)	303	157	0,87
North-East	402 (20.6%)	257	145	
Central Italy	374 (19.17%)	241	133	
Southern Italy	467 (23.94%)	303	164	
Islands	248 (12.71%)	168	80	
Residence (population density) (n = 1951)				
Rural (< 20.000 inhabitants)	863 (44.23%)	581	282	0,033
Town (20.000–100.000 inhabitants)	595 (30.5%)	362	233	
City (100.000–500.000 inhabitants)	200 (10.25%)	127	73	
Metropolis (> 500.000 inhabitants)	293 (15.02%)	202	91	
Parental origin (*n* = 1940, 10 missing)				
Both Italians	1626 (83.77%)	1055	571	0,3
Italian mother, non-Italian father	47 (2.42%)	30	17	
Italian father, non-Italian mother	92 (4.74%)	58	34	
Both non-Italians	175 (%9.02)	125	50	
Family composition (*n* = 1941, 9 missing)				
Living with both parents	1616 (83.21%)	1036	580	0,012
Living with the mother	198 (10.20%)	134	64	
Living with the father	21 (1.08%)	18	3	
Living with mother and stepfather	90 (4.63%)	71	19	
Living with father and stepmother	5 (0.26%)	2	3	
Living without the parents	11 (0.57%)	6	5	

### Headache prevalence

A total of 1272 adolescents (566 males and 706 females) affirmed to suffer or have suffered from headache at least several times in a year, accounting for 65.9% of life-time prevalence in the overall study population. In 38 % (38.8%) of the cases, headache lasted usually 2 hours or more. The mean frequency of headache was as follows: > 1/week (9.84%, *n* = 190), > 1/month (14.25%, *n* = 275), once a month (24.15%, *n* = 466), less than monthly (17.67%, *n* = 341), never or very rarely (less than 5 episodes lifelong) (34.09%, *n* = 658).

Mean age at headache onset was 8.4 years. In 18.33% headache episodes started before the age of six, in 55.3% between six and ten, and in 26.34% at the age of 11 or later.

About one-half of the students reported a positive family history for headache (46.73%).

### Headache characteristics

Further headache characterization was accomplished taking only into consideration Group 1 (Table 2), in which headache episodes occur with variable frequency. Most patients in Group 2, reporting very occasional or no episodes of headache at all, did not complete this questionnaire section. The mean duration of a single headache episode was less than 30 minutes in 32.9%, 1 hour in 28.12%, 2 hours in 19.33% and several hours in 19.49%. With respect to pain intensity, the results were the following: 38.3% mild, 52.2% moderate and 9.5% severe. Nearly half of the participants (42.5%) stated that there was no need to suspend their activities during a headache attack, while 36.3% needed to stop them sometimes, and respectively 10.9 and 10.3% needed to suspend their daily activities systematically or must seek for bed rest. Headache localization was more commonly fixed (50%); 24.6% students stated that localization was variable in time, and 25.4% could not determine a precise localization.

**Table 2:** Headache characteristics^a^

Characteristic	Boys	Girls	Total	Statistics	*p*-value
Age at Onset (*n* = 220)	7.98 (2.73)	8.69 (2.62)	8.33 (2.69)	W = 12,933	0.058
Family History (*n* = 1250)					
Yes	254 (45.4%)	375 (54.3%)	629 (50.3%)	χ^2^ = 9.64	0.002
No	305 (54.6%)	316 (45.7%)	621 (49.7%)		
Headache duration (*n* = 1251)					
< 30 min	203 (36.4%)	210 (30.3%)	413 (33%)	χ^2^ = 6.8	0.079
About 1 h	148 (26.6%)	204 (29.4%)	352 (28.1%)		
About 2 h	96 (17.2%)	146 (1.0%)	242 (19.3%)		
> 2 h	110 (19.7%)	134 (19.3%)	244 (19.5%)		
Intensity (*n* = 1257)					
Mild	243 (43.5%)	239 (34.2%)	482 (38.3%)	χ^2^ = 12.65	0.002
Moderate	272 (48.7%)	384 (54.9%)	656 (52.2%)		
Severe	43 (7.7%)	76 (10.9%)	119 (9.5%)		
Pain Localization (*n* = 1261)					
Localized at fixed points	279 (49.6%)	352 (50.4%)	631 (50.0%)	χ^2^ = 1.1	0.59
Different localizations	133 (23.7%)	177 (25.3%)	310 (24.6%)		
I don’t know	150 (26.7%)	170 (24.3%)	320 (25.4%)		
Need to stop daily activities (*n* = 1256)					
No	269 (48.2%)	265 (38.0%)	534 (42.5%)	χ^2^ = 13.95	0.003
Sometimes	179 (32.1%)	277 (39.7%)	456 (36.3%)		
Always	59 (10.6%)	78 (11.2%)	137 (10.9%)		
Always, and I need to rest in bed	51 (9.1%)	78 (11.2%)	129 (10.3%)		
Moment of the day (*n* = 1260)					
During the day	227 (40.5%)	337 (48.1%)	564 (44.8%)	χ^2^ = 12.3	0.015
At night	38 (6.8%)	30 (4.3%)	68 (5.4%)		
Both during the day and at night	84 (15.0%)	111 (15.9%)	195 (15.5%)		
At fixed hours	24 (4.3%)	18 (2.6%)	42 (3.3%)		
I can’t identify a specific timing	187 (14.8%)	204 (29.1%)	391 (31.0%)		
Seasonality (n = 1261)					
Spring	37 (6.6%)	53 (7.6%)	90 (7.1%)	χ^2^ = 6.9	0.137
Winter	92 (16.4%)	147 (21.0%)	239 (19%)		
Fall	24 (4.3%)	27 (3.9%)	51 (4.0%)		
Summer	44 (7.8%)	39 (5.6%)	83 (6.6%)		
No differences	365 (64.9%)	433 (61.9%)	798 (63.4%)		
Difference between school and holiday (*n* = 1259)					
No	233 (41.6%)	219 (31.3%)	452 (35.9%)	χ^2^ = 20.8	< 0.001
More during school	307 (54.8%)	468 (67.0%)	775 (61.6%)		
More during holiday	20 (3.6%)	12 (1.7%)	32 (2.5%)		
Can you identify a trigger factor? (n = 1261)					
Yes	361 (64.3%)	528 (75.4%)	889 (70.5%)	χ^2^ = 18.38	< 0.001
No	200 (35.7%)	172 (24.6%)	372 (29.5%)		

^a^in Group 1

**Fig. 1:**
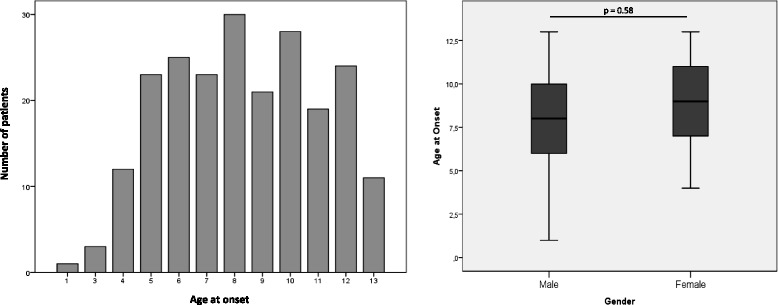
Mean age at headache onset (left) and gender differences in age at headache onset (right). The age distribution at headache onset shows two peaks in peri-pubertal age, at 8 and 10 years, with a mean age at onset of 8.3 years and an overall increase during adolescence. Girls show a moderate tendency for older age at onset, without reaching statistical significance

Mean age at headache onset in Group 1 was 8.33 years. Age distribution at headache onset is illustrated in Fig. 1.

Headache was reported to occur during the day in 44.8%, while 15.5% experienced headache invariably during the day and the nighttime and 31% was unable to identify a very moment of onset. The most common season associated to headache exacerbations was winter (19%), followed by spring (7.1%) and summer (6.6%) although most of the students did not note any difference in seasonality (63.4%). Headache was far more frequent during the school period than during the holidays (61.6%).

**Fig. 2:**
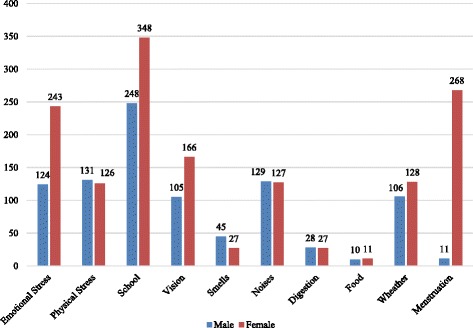
Self reported trigger factors for headache and gender differences. School and study represent the main trigger factors for headache for both sexes during adolescence. Menstruation and emotional stress constitute the principal triggers for girls, while males tend to consider loud noises, weather change and emotional stress as major provoking factors for headache. Significantly more girls than boys could identify a definite trigger (75.4% vs 64.3%, *p* < 0.001)

Considering trigger factors, students usually managed to find a cause. Triggers were selected by the students from a list which comprised the principal pediatric headache-related provoking factors reported in the literature [18–21], with the possibility to add additional triggers if not included. The reported triggers, in decreasing order, were the following: school or study (67%), emotional stress (41.3%), menstruation (31.4%), vision (30.5%), physical stress (28.9%), loud noises (28.8%), weather change (26.3%), smells (8.1%), digestion (6.2%), specific food (2.4%) (Fig. 2).

### Medication use

Considering the general study population, more than two thirds of the adolescents (69.15%) reported the use of medication for headache control. When considering Group 1, this percentage rises to 73.9%. A rate of 16.8% reports the use of analgesics systematically, whereas 57.2% resort to medication only in case of moderate or severe pain. A small percentage of students use pain relievers at the beginning of a headache episode (7.4%), while a greater part wait until the pain is moderate/severe (55.5%) or until it is unbearable (37.1%) before taking drugs.

To the question “How do you decide which type of medicine to take?” the majority answers “I ask to my parents” (67.2%), while only a few follow a physician’s prescription (20.6%) or a pharmacist’s advice (6.5%). Up to 5.7% report self-medication, autonomously deciding which drug to take.

In choosing the best medication, a big importance is given to the power of the drug in terms of rapidly relieving the pain (66.5%), while only a minority of adolescents do primarily care about its long-lasting efficacy (16.3%) or the absence of side effects (17.2%). The majority of the investigated teenagers reported no fears about painkillers (44.5%), while 17.2% stated not to have even thought about the problem before, and only 38.3% affirmed to avoid their use as much as possible.

Medication use among male and female students in Group 1 is illustrated in Table 3.

**Table 3:** Therapy^a^

	Boys	Girls	Total	Statistics	p-value
Takes medication (n = 1259)					
Always	89 (15.9%)	122 (17.4%)	211 (16.7%)	χ^2^ = 23.7	< 0.001
Only for severe pain	287 (51.3%)	433 (61.9%)	720 (57.2%)		
Never	183 (32.7%)	145 (20.7%)	328 (26.1%)		
Worries about medication use (*N* = 1223)					
No	287 (53%)	257 (37.7%)	544 (44.5%)	χ^2^ = 31.58	< 0.001
Yes	187 (34.5%)	282 (41.4%)	469 (38.3%)		
Never thought about it	68 (12.5%)	142 (20.9%)	210 (17.2%)		
How do you decide which medication to take (*n* = 1089)					
Prescribed by doctor	113 (24.1%)	111 (17.9%)	224 (20.6%)	χ^2^ = 10.34	0.016
Ask parents	294 (62.8%)	438 (70.5%)	732 (67.2%)		
Ask pharmacist	37 (7.9%)	34 (5.5%)	71 (6.5%)		
I decide what to take	24 (5.1%)	38 (6.1%)	62 (5.7%)		

^a^in Group 1

### Gender and age-related differences

A significant difference was seen between females and males: the former experiencing headache more frequently (70.2% females vs 60% males in group 1, *p* < 0.001) and more intensely (40.5% vs 52.6% mild, 50.3% vs 41.1% moderate and 9.2% vs 6.3% severe headache, *p* < 0.001).

There was no significant difference in age at headache onset between boys and girls in Group 1 (mean age boys: 7.98 years; mean age girls: 8.69 years; *p* = 0.058) (Fig. 1). Further analyzing Group 1, we notice that girls have a statistically significant family history of headache (54.3% vs 45.4%, *p* = 0.002), they suffer more often than boys during school periods (67% vs 54.8%, *p* < 0.01) and more during the daytime (48.1% vs 40.5%, *p* = 0.015). Moreover, adolescent girls more frequently need to stop their activities (sometimes: 39.7% vs 32.1%, always: 11.2% vs 10.6%, *p* = 0.003) or even go to rest in bed in case of headache (11.2% vs 9.1%, *p* = 0.003), while male peers are clearly less affected into their everyday functions (Table 2). Girls are more able to identify trigger factors than boys (75.4% vs 64.3%, *p* < 0.001). Menstruations are the second main trigger in females, after school and study. Trigger types also seem to differ between boys and girls: the former report physical stress, loud noises and smells more frequently (respectively 14% vs 8.6%, 13.8% vs 8.6% and 4.85% vs 1.8%; *p* < 0001), while emotional stress plays a major role in females (16.5% vs 13.2%; *p* < 0.001) (Fig. 2). All these results were statistically significant.

Significantly more boys than girls report never taking medication for headache control (32.7% vs 20.7%, p < 0.001), and having no worries about taking painkillers (34.5% vs 41.4%, *p* < 0.01). Both sexes preferentially ask parental advice in choosing the adequate medication (70.5% of females vs 62.8% of males, *p* = 0.016), but girls more often report self medication (6.1% vs 5.1%) and state they never thought about medications side-effects (20.9% vs 12.5%, p < 0.001), while boys more often follow a physician’s prescription (24.1% vs 17.9%, p = 0.016).

No significant difference was seen in age distribution regarding headache prevalence, headache frequency and pain intensity.

### Geographic and demographic factors

There was no significant difference in headache prevalence among different geographic regions. Students living in the North-West and in the Islands are more likely to suffer from headache for several hours (23.5% North West, 21.9% Islands, 18.5% South, 18.1% North-East, and 15.6% Center, *p* = 0.031), and with a severe intensity score when compared to the other regions (14.3% North West,11.7% Islands, 6.6% North East, 5.4% Center, and 9.1% South, *p* = 0.003).

We observed no regional difference concerning the use of painkillers, timing and self-medication recourse. In Southern Italy and Islands, significantly more adolescents take medication according to a physician prescription, while students from the North-East more frequently rely on parental advise (physician’s advice: 28.3% Islands, 25.5% South, 19.6% Center, 17.5% North West, and 14.1% North East, parental advice: 75.9% North East, 67.6% Center, 66.5% North West, 63.4% Islands, and 62.4% South, *p* = 0.02). Students from the North West less frequently stated to be worried of potential side-effects and risks of medication than southern peers (30.2% North West, 38.6% North East, 40.9% Islands, 41.3% South, 43% Center, *p* = 0.027). Only these latter results, concerning duration and severity of headache, medication use and medication-related worries, maintained statistical significance after grouping this data into a north-to-south gradient (North-West and North-East; Central Italy; South and Islands). We had, respectively: *p* = 0.012, *p* = 0.017, p = 0.017, and *p* = 0.049.

Population density does not seem to influence headache characteristics, use of painkillers, timing and self-medication. Nevertheless, adolescents living in big metropoles or rural areas show a higher headache prevalence compared to those living in towns and cities (respectively 68.9% and 67.3% vs 60.8% and 63.5%; *p* = 0.033) (Table 1).

We found no significant difference in sex distribution and seasonality according to geographic and demographic factors.

### Social and familial factors

Children living with both parents are less likely to suffer from headache in comparison with children living with only one parent, or without parents (64.1% vs 70.4%, *p* = 0.027). Similarly, pain intensity is greater in those who are not living with both parents compared with those who do (respectively 36.6% vs 38.7% mild pain, 49.1% vs 52.9% moderate pain, and 14.2% vs 8.4% severe pain; *p* = 0.023). Family composition did not significantly influence medication use in our population.

Data were further analyzed to determine whether the birth place (Italy or abroad) and parental origin could influence the epidemiology of headache in our population: no statistical differences were found in prevalence and pain intensity. Students born in Italy, however, tend to use painkillers later in case of headache (56.1% vs 47.1%; *p* < 0.01), and more frequently state to have no worries about taking medications (43.6% vs 58.3%; *p* = 0.022).

## Discussion

Sixty-five percent of adolescents suffered from headache in our study population, with a female to male sex ratio of 1.25:1. Headache prevalence greatly varies among different studies in childhood, as it can be influenced by both methodological (i.e. 6-months, one-year or lifetime prevalence; population-based or clinical based studies; diagnostic and selection criteria) and demographic factors (i.e. age and sex distribution, socio-economic factors) [3, 6, 7, 9, 22, 23]. Similar lifetime prevalence and sex ratio have been reported in recent population-based studies on headache and migraine among adolescents [2, 8, 13, 14, 24–26]. Population-based studies are known to detect a significantly higher prevalence of headache among adolescents: this is particularly true for those based on self-reporting questionnaires, as parents tend to underestimate the real burden of headache in this age group [13].

The age of symptoms onset in our study showed two peaks in peri-pubertal age, at 8 and 10 years, with a mean age at onset of about 8.3 years and an overall increase of headache onset with age (Fig. 1). This is in line with the results of recent Italian multicenter and single-center studies [3, 11], and several international publications showing an increment of the lifetime prevalence of headache from 47.2% in children 7–9 years old to 69.5% in those 13–15 years old [15, 27–29]. Other studies have shown a later onset [7, 30], but this is probably attributable to different geographical and methodological factors. In their population-based study in Croatian adolescents, Cvetković and colleagues evidenced a significant older age at headache onset in girls with respect to male peers [7]. In our data, girls showed a moderate tendency for older age at onset without reaching statistical significance, possibly because of the small sample (only 220 students specified their age at onset in Group 1).

Genetic predisposition in headache and migraine is well documented [31, 32]. In the series by Kroner-Herwig et al., children of parents suffering from headache were also sufferers in 72% of cases, whereas in families where parents were headache-free only 27% of the children were diagnosed with headache [10]. In our study, a familiar predisposition was reported in 50.3% of students in Group 1, but this rate was significantly higher in females (54.3% vs 45.4%). Isense et al. suggest that in recognizing parental headache as a risk factor, not only genetic predisposition should be considered, but also the influence of dysfunctional pain-related modelling mechanisms: in this perspective, parental perception of and coping with headache may very soon influence the child’s cognitive, emotional and behavioral coping with pain, influencing intensity, frequency and pain-related disability [33].

Our results support recent findings that special consideration should be given to female adolescents [33, 34]. Other studies have revealed a higher incidence of headache as well as a higher burden, clinical heterogeneity and higher psychological comorbidity such as depression and anxiety in girls as compared to boys [6, 13, 33, 35]. However, this sex discrepancy seems to become relevant only from adolescence on [4, 6, 10, 36]. This study confirms a higher headache prevalence and intensity score in adolescent girls. Moreover, girls suffer significantly more often than boys during the school period and more frequently need to stop their activities in case of headache: this suggests a higher impact on their daily functioning and quality of life. Interestingly, girls seem more able than boys to identify trigger factors for headache exacerbations. 18.2% of girls identified menstruations as the major trigger in our study. Together with school issues and emotional stress, these accounted for 58.4% of all triggers in females. Finally, adolescent girls take more analgesics and recur to self-medication more frequently than male peers, while only 17.9% of them are treated following a physician’s advice. Analogous results have been reported in past studies [7, 30]. This findings may be essential to delineate better prevention strategies and underline the importance of promoting behavioral therapies, environmental hygiene and comorbidities management in adolescents with headache, particularly in girls. They also should rise physician’s awareness of the problem and encourage population screenings and timely adapted therapeutic approaches among health care professionals.

Several environmental factors are known to play a role in headache pathogenesis. Although the majority of subjects did not note seasonality changes in headache, winter was fairly the season most associated to headache exacerbations in those who did. In almost two thirds of the cases, headache occurs during school period and school/study represents the most common reported trigger factor in both sexes, followed by emotional stress. The literature reports a wide relationship between school issues and headache frequency, duration and intensity: notably, headache appears to be related to worries about performance, marks and promotions [3, 30].

Adolescents with migraine report significant impairment in health-related quality of life compared to non-migrainous peers, independently from psychiatric comorbidities [37]. The literature also reports numerous associations between headache frequency and severity, and several diverse physical complaints as well as physical and psychiatric morbidity later in life [10, 16, 38–40]. This is particularly true for those children with high headache severity, who report the lowest quality of life, as well as the most issues regarding physical functioning, impact on daily and leisure activities and social functioning [41]. Furthermore, chronic conditions such as migraine are associated with a substantial burden on the cohabitating family members, who report a significant effect on family life and social activities [42]. Although we did not specifically consider psychiatric comorbidities in this study, our results suggest that more than half of the adolescents variably recognize an impact on daily activities during headaches attacks. Among those, nearly a half need to give up daily activities or seek for bed rest until the symptoms are over. Similarly, in a recent study assessing headache among adolescents in Croatia, 29.7% stayed at home and needed bed rest because of headache [7].

Headache during childhood has a high risk of persisting into adulthood [43]; it is therefore important to give adequate management to adolescents with recurrent and severe headaches. The majority (73.9%) of students suffering from headache takes medications for symptoms control: our data are in line with other studies, in which medication use is reported in a variable percentage going from 30% to 80% [3, 7, 30]. Nonetheless, only 16.7% reports systematical use of analgesics, while the majority waits until the intensity becomes moderate or high. While the larger part of adolescents ask their parents for medication advise, the percentage of those reporting self medication is considerable (5.7%). Overall, only the 20.6% declared to take medications following a physician’s prescription. This was reported respectively in 22.6 and 31.1% in different studies by Poyrazoglu [30] et al. and Kröner-Herwiget et al. [10], while it rises to 89% when taking into account specialized clinical settings based on tertiary centers for headache management [11]. Although it may be interpreted as a health behavior (relying on the child’s own capability to cope with the problem), the fact that the majority of students experiencing recurrent headache is treated without medical advice may underlie a parental underestimation of the headache burden. This may finally lead to undertreatment, or possibly encourage self-medication with the risk of misuse or abuse of painkillers [44].

Strengths of our study include the big sample-size and a low drop-out rate. Our study population was highly representative of the Italian adolescent population, as classes were randomly selected from secondary schools considering geographically and population-density data provided by the Italian Ministry of Education. A school-based population study eliminates the risk of selection-bias that is common in studies conducted in clinical settings. An anonymous, multiple-choice questionnaire allows a sincere and prejudice-less investigation in a by definition “fragile” population. Adolescents may frequently feel uncomfortable about talking about personal physical and psychological issues with medical or paramedical professionals: in this sense, an anonymous questionnaire may consistently minimize over- and under-estimations.

Our study has some limitations: 1) headache intensity and frequency was very heterogeneous in our study population, ranging from episodic attacks to chronic headache. 2) Headache was not classified following the ICHD criteria. This constitutes a major limit both for the lack of distinction between different types of primary headache, and between primary and secondary headache. Such classification requires validation by a trained specialist along with a throughout anamnestic investigation, and would not have been possible in the context of a self-evaluation questionnaire-based study. Moreover, it is difficult to categorize the type of headache in children because of the significant overlap of symptoms that meet both criteria for migraine without aura and tension-type headache, and this may frequently lead to misinterpretations [45]. A recent study by Balestri et al. underlined that classification of headache could be difficult considering duration criteria as a fundamental key [46], and Torriero et al. reaffirmed this statement suggesting that the exclusion of headache duration criteria allowed a diagnosis of primary headache in more than 80% of children in their study [47]. Furthermore, Ozge and colleagues recently commented on the necessary distinction between pediatric and adult headache, emphasizing that specific subtopics of pediatric headache should be carefully defined and included in next ICHD criteria [48]. 3) All questionnaire-based studies have intrinsic limitations, related to recall and interpretation biases. In this sense, students may have differently understood the questions and replied based on own interpretations with inadequate objectivity. Nevertheless, the questionnaire was completed under the supervision of a trained teacher, and students were encouraged to ask for advice and explanations to avoid misunderstandings. 4) Finally, no validated standardized questionnaire was given to determine quality of life, which was indirectly deduced from answers regarding impact on daily activities.

## Conclusions

In this study we aimed to determine prevalence and characteristics of headache in a nation-wide representative sample of adolescents, a population which remains widely reported. Our results indicate that adolescence is at high risk for headache disorders, and support the need for more attention from health authorities and professionals: lifetime headache prevalence is high (reaching its peak in peri-pubertal age), underdiagnose and undermanagement are common and self-medication is frequent, while trigger factors are often easily detectable (thus targetable for specific primary and secondary prevention interventions). Adolescent girls, in particular, seem to carry the major medical and social disease burden. It is essential to offer adolescents who suffer from headache a timely psychological and medical management in order to implement quality of health and quality of life, and prevent chronic headache later in life.
